# An Investigation of the Role of Ridden Exercise, Including Application of the Ridden Horse Pain Ethogram, in Pre-Purchase Examinations of Sports Horses

**DOI:** 10.3390/ani16132105

**Published:** 2026-07-07

**Authors:** Sue Dyson

**Affiliations:** Independent Researcher, The Cottage, Church Road, Market Weston, Diss IP22 2NX, UK; sue.dyson@aol.com

**Keywords:** lameness, canter dysfunction, poor performance, saddle slip, teeth grinding, behaviour

## Abstract

Many horses undergo a pre-purchase examination (PPE) prior to acquisition by a new owner; however, despite evidence that lameness and canter dysfunction may only be apparent during ridden exercise, ridden exercises assessing gait quality is not a routine part of PPEs. The objectives were to determine the influence of the results of ridden exercise on decision-making concerning risk assessment for purchase and to establish the long-term performance of horses. Twenty-five sports horses underwent PPEs including ridden exercises to assess gait quality and to apply the Ridden Horse Pain Ethogram (RHpE). Follow-up information concerning performance was acquired from British Eventing, British Dressage, and British Showjumping. Eight horses were considered a reasonable risk for purchase, none of which showed lameness during ridden exercises. All had RHpE scores ≤ 4/24. Six of these horses fulfilled the purchaser’s expectations, all competing at a higher level than previously. One horse sustained a freak accident necessitating humane destruction. One horse developed lameness. Three horses with RHpE scores of 3 or 4/24 were considered suitable, but with reservations. None were purchased, and only one competed after an 18-months’ interval. The remaining horses were considered high risk, and the twelve that were ridden had a median RHpE score of 6/12 (interquartile range 5–7, range 4–16). Of these, six horses showed overt lameness when ridden and six had gait dysfunction. Only one horse continued in competition; three horses returned to competition after six to eighteen months. The study included only 25 horses evaluated over six months; however, this facilitated acquisition of accurate long-term follow-up information. In conclusion, there were benefits of evaluating ridden exercise, including the application of the RHpE and identification of lameness or canter dysfunction, during PPEs.

## Simple Summary

In most countries, ridden exercise is not part of a routine pre-purchase examination (PPE), or ridden exercise is performed to assess the physiological response to strenuous exercise rather than to evaluate the quality of gaits. However, there is evidence that a horse may be non-lame in hand and on the lunge but show gait dysfunction when ridden. The purposes of this study were to document clinical findings during static examination and during dynamic examinations, including walk and trot in-hand, lungeing, and ridden exercise; to determine Ridden Horse Pain Ethogram scores; and to compare the results with subsequent competition performance over the following four years. The results of 25 consecutive PPEs over six months were reviewed. Horses were classified as a reasonable risk for purchase, suitable but with reservations, or high risk. Evaluation of horses during ridden exercise, assessing both gait and behaviour, gave valuable additional information to enhance the accuracy of risk assessment for the purchaser’s proposed use of the horse. Most horses with gait dysfunction and/or multiple abnormal behaviours during ridden exercise did not return to competitions run under the jurisdiction of British Eventing, British Showjumping, or British Dressage.

## 1. Introduction

Many horses undergo a pre-purchase examination (PPE) prior to acquisition by a new owner, but there are no uniform global guidelines as to how PPEs should be performed or reported. There is a growing body of evidence documenting that lameness or other gait dysfunction may not be recognised in hand or on the lunge but are evident during ridden exercise, or that minor gait abnormalities seen in hand are accentuated during ridden exercise [1,2,3,4,5,6,7,8]. Purchasers of a horse usually want a riding horse. It therefore appears logical that ridden exercise should be part of PPEs. Ridden exercise is part of a PPE in the United Kingdom and Australia nominally to assess the physiological response to strenuous exercise, but it has not been mandated to specifically evaluate either gait normality and quality or equine behaviour. Ridden exercise is not routinely included in PPEs in North America, South Africa, and many European countries.

It appears that reliance is placed, perhaps erroneously, on a rider and/or coach having previously assessed a horse’s ridden performance as suitable before instructing a veterinarian to perform a PPE. However, there is a wealth of evidence that riders [1,2,3,4,5,6,7,8,9,10] and coaches [11] are both poor at recognising lameness. Moreover, they have limited knowledge or awareness of abnormal behaviour during tacking-up and mounting [12,13] and during ridden exercise [14] as a potential manifestation of musculoskeletal pain. It therefore seems logical that ridden exercise, to assess both gait quality and behaviour, should be part of a PPE.

There is evidence that the quality of gaits can be improved by a skilled rider compared with a less skilled rider [15], but the behavioural signs of discomfort cannot be concealed. It is a common observation that vendors blame a potential purchaser for a less than perfect ride when trying a horse because of their limited skills, which could in part be true, but underlying musculoskeletal pain may be a contributing factor.

The Ridden Horse Pain Ethogram (RHpE) was developed to facilitate the recognition of musculoskeletal pain and has been proven to be a useful tool [16,17,18,19,20]. Most sports horses without musculoskeletal pain have RHpE scores of 0–4/24 [16,21,22,23,24,25,26]. A threshold score of ≥8/24 indicates the likely presence of musculoskeletal pain, although some lame horses score less than 8. High RHpE scores have been associated with reduced performance in dressage and eventing [21,22,23,24,25]. It seems likely that the use of RHpE may facilitate decision-making following PPEs if horses are evaluated while being ridden.

There are no previous studies that have investigated the long-term performance outcome of horses which have undergone PPEs. This study aimed to utilise performance outcomes to determine the accuracy of risk assessments by calculating sensitivity, specificity and accuracy of the predictions. In this context, sensitivity is the ability of the PPE to correctly identify horses that will *ultimately succeed* in competing at the same level or higher than prior to purchase. Specificity is the ability of the PPE to correctly identify horses that will *not succeed* (i.e., ruling out unsuitable candidates to prevent purchasing a horse that will fail or require excessive management/veterinary care).

There is a trend to perform multiple imaging studies in association with PPEs rather than focus on the clinical presentation of the horse. Images provide a historic record of musculoskeletal injury and findings may or may not be of current or future clinical significance. A comprehensive assessment of the whole horse and its current performance, including during ridden exercise, may be of greater relevance. The purpose of the current study was to determine the influence of the results of ridden exercises during PPEs on decision-making concerning purchase and to establish the long-term performance of horses when available.

## 2. Materials and Methods

The results of 25 PPEs performed consecutively by the author over a period of six months were reviewed. All vendors and purchasers gave informed consent for data to be used anonymously for research purposes. All data were stored in alignment with General Data Protection Regulation guidelines. Follow-up information, when available, was determined over 3.5–4 years after the PPE by the review of clinical records and official competition websites (British Showjumping [BS], British Eventing [BE], and British Dressage [BD]). If a horse did not appear in these databases, it can be assumed that it is unlikely that the horse competed at official competitions, unless it had undergone a name change, which requires payment of substantial fees to the original Passport Issuing Organisation and the corresponding ruling sports organisation.

All examinations were performed in compliance with the Joint Memorandum of the British Veterinary Association and the Royal College of Veterinary Surgeons, including the options of distal forelimb flexion tests (each performed for one minute); proximal hindlimb flexion tests (each performed for one minute); lungeing on a soft surface at trot and canter (15–20 m diameter circle); and lungeing on a firm, non-slip surface at trot (15–20 m diameter circle) and ridden exercise. Horses were evaluated as tacked-up and mounted before being ridden in an arena or field and observed at walk, trot (rising and sitting), and canter, including 20 m and 10 m diameter circles in rising trot on the left and right reins, as well as repeated transitions between gaits. Horses were also assessed performing additional movements, such as shoulder-in, half-pass, and canter–flying changes, as required by the work discipline and level at which a horse was training and competing. Horses were usually ridden by the vendor (or their agent) but sometimes also by the purchaser.

Abnormalities of behaviour during tacking-up and mounting were recorded [12,13]. Static saddle fit for the horse was assessed before ridden exercise [27]. Saddle slip (the saddle consistently slipping to one side) was recorded as present or absent [2,28]. Lameness was graded and recorded independently for every circumstance in which each horse was examined (0 = non-lame, 1 = just discernible, 2 = mild, 3 = mild–moderate, 4 = moderate, 5 = moderate–severe, 6 = severe, 7 = partially weightbearing, 8 = non-weightbearing), together with descriptions of any abnormalities of the gait in walk, trot, and canter [29]. Canter dysfunction was defined as one or more of the following: unwillingness to establish canter; repeated strike off with the incorrect forelimb leading; short stepping; stiffness; on the forehand; close temporal and spatial placement of the hindlimbs; dissociation of placement of the trailing forelimb and the leading hindlimb (4-beat canter); lack of a suspension phase; becoming disunited repeatedly (cross-cantering); breaking spontaneously from canter to trot repeatedly; or bucking in trot–canter transitions and/or in canter [4,29]. The RHpE was applied during ridden exercise in real time. The presence of teeth grinding or repeated chomping on the bit was documented.

Prior to the PPEs, the horse’s competition record was reviewed if applicable. The purpose for which the horse was being purchased was determined to include the level of anticipated training/competition. Purchasers were generally present at PPEs, so that observations could be discussed in real time and, when applicable, the examination could be terminated prematurely. There was a final discussion about the horse’s suitability for purchase at the end of the examination. Radiography ± ultrasonography of selected regions, guided by the purchaser, were performed if, based on the clinical assessment, the horse was considered a potentially suitable risk for purchase. In none of the horses did the results alter the outcome; therefore, imaging findings are not reported. Purchasers were advised that a horse was suitable for its intended purpose (reasonable risk), suitable but with reservations, or that the problems identified represented a high risk for purchase.

The data are described using descriptive statistics. With the outcome data, success was defined as continuing at the same level of competition as pre-sale or higher. The ‘Reasonable Risk’ group (excluding the horse which was humanely destroyed) versus the higher risk groups were compared using Fisher’s Exact Test. The ‘number needed to treat (identify as high risk)’ was calculated [30]. The diagnostic accuracy of the PPE findings was assessed by calculating sensitivity, specificity and accuracy, comparing the rate of horses continuing to compete, which were classified as ‘Reasonable Risk’, versus those categorised as higher risk, with results presented with Clopper–Pearson Confidence Intervals (CIs) (https://www.medcalc.org/en/calc/diagnostic_test.php [Version 23.5.8; accessed 31 May 2026]). A true positive (TP) result was when a horse was deemed to be a reasonable risk at the PPE and went on to compete. A true negative (TN) result was when a horse was deemed as a high/intermediate risk and failed to compete. A false positive (FP) result was when a horse was deemed a reasonable risk but failed to compete. A false negative (FN) result was when a horse was deemed as a high/intermediate risk but went on to compete. Sensitivity, specificity and accuracy were calculated as follows: Sensitivity = TP/(TP + FN); Specificity = TN/(FP +TN); Accuracy = Sensitivity × Prevalence + Specificity × (1 − Prevalence).

Statistical significance was set at *p* < 0.05.

## 3. Results

### 3.1. Horse Details

There were seven mares and 18 geldings, ranging from 4 to 12 years of age (median 6 years, interquartile range [IQR] 5–8) (Table 1). There were 15 Warmbloods, four Irish Sports Horses, two Thoroughbreds, three crossbreeds, and one pony. The intended use of the horse was dressage (*n* = 6), showjumping (*n* = 3), or eventing (*n* = 16) under the auspices of the official governing bodies (BD, BS, and BE). Purchasers of 24 horses had intentions that the horse should train and compete at a higher level than previously. The single event pony (H23) had competed successfully internationally. All purchasers were amateur riders, except one professional rider (H15) buying on behalf of a sponsor. One purchaser (H10) wished to be able to insure the horse for veterinary fees without any exclusions on the policy.

Two horses were not evaluated ridden. Horse 24 resented distal limb flexion of the right forelimb, exhibited a sustained grade 5/8 right forelimb lameness after distal limb flexion, and showed grade 2/8 right forelimb lameness on the left and the right reins on the lunge on both soft and firm surfaces. Horse 25 exhibited grade 1/8 left forelimb lameness in hand, increasing to grade 2/8 on the left rein on the lunge on both soft and firm surfaces, and showed grade 4/8 right forelimb lameness on the right rein. Horse 24 was not purchased, did not compete for six months, and then resumed competition for two years, scoring 67–70% at Novice (United States Equestrian Federation [USEF] 1st level) and Elementary (USEF 2nd level) dressage levels; however, it has not competed for more than one year since. Horse 25 was not purchased and was lost to follow-up (i.e., did not compete under BD, BE, or BS rules). These two horses are not discussed further but were included to demonstrate the spectrum of outcomes in the consecutive PPEs performed over six months.

### 3.2. Static Examination

The results of the static examinations are summarised in Table 1. Five horses (H1, 6, 9, 20, 21) had underdevelopment of the thoracolumbosacral epaxial musculature relative to the level at which they had allegedly been working. Eight horses (H2, 11, 14, 16, 20, 21, 22, 25) had thoracolumbosacral epaxial muscle hypertension and/or an abnormal response to pressure applied over the tubera sacrale, seven of which (87.5%) were ultimately considered a high risk for purchase. Seven horses (H1, 6, 7, 10, 12, 14, 20) had mild asymmetry of the pelvic musculature.

### 3.3. Dynamic Examination in Hand

The results of the dynamic examinations are summarised in Table 2. During in hand examinations, one horse (H14) had a bilateral hindlimb toe drag, five horses (H15, 19, 20, 22, 23) appeared to be short stepping, and five horses had grade 1/8 (H6, 7) or grade 2/8 (H10, 17, 21) unilateral hindlimb lameness. One horse (H13) exhibited subtle forelimb and hindlimb ataxia.

### 3.4. Dynamic Examination on the Lunge

Four horses (H3, 12, 15, 17) showed forelimb lameness ≤ 3/8 on the lunge (Table 2). Seven horses (H11, 13, 14, 20, 21, 22, 23) exhibited poor hindlimb impulsion and engagement in trot, while seven horses (H3, 4, 9, 11, 14, 21, 23) had canter dysfunction.

### 3.5. Tacking-Up and Mounting

Five horses (H1, 9, 11, 16, 21) showed abnormal behaviours when tacked-up [12,13], ranging from repetitive teeth grinding (H1, 16 [teeth grinding continued throughout ridden exercise]) to aggressive behaviour and trying to escape from the cross-ties (H21). Horses 16 (flexed the thoracolumbar region, ‘humped the back’) and 21 (‘yanked down’ with the head and neck repeatedly) also showed abnormal behaviour when mounted, in addition to H15 (extended the thoracolumbar region, ‘dipped the back’).

### 3.6. Ridden Exercise

#### 3.6.1. Gait Quality

Three horses, which showed low-grade forelimb (H3) and hindlimb (H6, 7) lameness in hand, did not show lameness when ridden and had RHpE scores of 2/24 (H3, 7) and 3/24 (H6) (Table 2). Neither lameness nor canter dysfunction were detected in seven horses (H1, 2, 5, 6, 10, 16). However, H16 had poor quality transitions, a reduced range of motion of the thoracolumbosacral region in sitting trot compared with rising trot, repeatedly ground its teeth, and its RHpE score was 7/24.

Lameness became apparent during ridden exercises or was worse than previously reported in six horses (H12, 14, 15, 18, 19, 21), five of which also had canter dysfunction. Lameness was similar to previous observations in eight horses (H8, 9, 11, 13, 17, 20, 22, 23), six of which also had canter dysfunction. Eleven horses were identified with canter dysfunction during ridden exercises compared with only seven during lungeing exercises, which represents a 36% increase during ridden exercises.

#### 3.6.2. Saddle Slip

Saddle slip occurred in six horses (H6, 11, 14, 15, 19, 20), five of which showed ipsilateral hindlimb lameness in ridden trot. Horse 6 had a grade 1/8 left hindlimb lameness in hand, which was not observed under other circumstance; the saddle slipped to the left.

#### 3.6.3. Teeth Grinding

Teeth grinding or repetitive chomping on the bit was observed in four horses (H1, 14, 16, 21), two of which had no detectable lameness (H1, 16), and two with hindlimb lameness (H14, 21).

#### 3.6.4. Stumbling

Two horses (H9, 17) that showed lameness in hand and when ridden stumbled badly at least once during ridden exercises.

#### 3.6.5. Ridden Horse Pain Ethogram Scores

Ridden Horse Pain Ethogram scores ranged from 1 to 16/24 (median 4, IQR 3–6).

### 3.7. Recommendations and Outcomes

Eight horses (H1–8) were recommended for purchase without major reservations. All had RHpE scores ≤ 4/24. Six of these horses subsequently fulfilled the purchaser’s expectations, all competing at a higher level than previously (Table 3). One horse (H2) sustained a comminuted articular fracture of the proximal phalanx of the right forelimb in a freak accident within three weeks of purchase and was humanely destroyed. One horse (H1), which showed teeth grinding during tacking-up and ridden exercise in the PPE, did not progress satisfactorily in training and developed bilateral hindlimb proximal suspensory desmopathy and lumbosacroiliac joint region pain. After successful treatment, the horse changed ownership and is used for general purpose riding. The horse’s movement quality and muscle development have improved substantially, and the horse no longer grinds its teeth (personal observation).

One horse (H9) was purchased despite reservations about its suitability and experienced chronic problems and was ultimately retired. Two horses were not purchased because of reservations: H10 (the insurance company would not provide cover without exclusions on the policy) and H11. H10 continued to be used for general purpose riding. Horse 11 did not compete for 18 months and then competed at advanced medium level dressage (USEF 4th level) scoring on average 70%.

Twelve horses (H12–23) were considered high risk and were not purchased, all with RHpE scores ranging from 4 (plus tension and saddle slip) to 16/24 (median 6, IQR 5–7). One horse (H20) continued to compete and progressed to Prix St Georges, with a mean score of 65%. Three horses (H15, 16, 22) did not compete again in affiliated competitions. Horse 16 underwent a PPE performed by another veterinarian on behalf of a different client three days after the initial examination and was purchased. However, the horse was returned to the vendor six weeks later because of dangerous behaviour during ridden exercise and did not compete thereafter. Horse 21 did not compete for one year and then competed for two years at medium (USEF 3rd level) and advanced medium levels, with a mean score of 66%. Horse 23 did not compete for one year and then was downgraded to BE 100 (under 18), competing four times and three times only in successive years, and then was further downgraded to BE 90, but failed to complete four events. Three horses (H17, 18, 19) were used for pleasure riding. Horse 14 was retired.

The ‘Reasonable Risk’ group had a significantly higher chance of a successful outcome than the higher risk groups (*p* = 0.0003), with an Odds Ratio of 96.0. The sensitivity for the ability of the PPE to correctly identify horses that would continue to compete was 87.5% (CI 42.1%, 99.6%), and the specificity for the ability of the PPE to correctly identify horses that would not compete was 94.1% (CI 71.3%, 99.9%). Accuracy, the overall probability that a horse was classified correctly, was 91.7% (CI 73.0%, 99.0%). The ‘Number Needed to Treat’ indicated that for every 1.1 horses advised against purchase, one significant performance failure was prevented.

## 4. Discussion

In most horses, the inclusion of ridden exercise provided useful additional information about each horse’s suitability for purchase. Three horses showing low-grade lameness in hand or on the lunge did not exhibit lameness when ridden and had low RHpE scores. These horses were recommended for purchase and performed satisfactorily thereafter. Ridden exercises revealed lameness and/or canter dysfunction associated with RHpE scores > 4/24 in more than half the horses examined. Most of these horses did not have successful subsequent competition careers. Overall, 32% of 25 horses were recommended for purchase. Only one horse (4%, H20), which was not recommended for purchase, continued in regular competition work. Three additional horses (H11, H21, H24) that were not recommended for purchase returned to competition at the same level as pre-sale after long (six to eighteen months) intervals.

Overall, 6/7 horses (86%) recommended for purchase (excluding the horse that was humanely destroyed following a freak accident) continued in regular competition work at the same level or higher than pre-sale for at least 3.5 to 4 years after purchase compared with only 1/17 horses (6%) that were considered at higher risk, although three additional horses (18%) eventually returned to competing at the same level.

The CIs for sensitivity and specificity were quite wide, especially for sensitivity, and the results of the statistical analyses must be interpreted both in this context and the small number of horses in the study. However, accuracy, the overall probability of the correct classification of a horse, was high (92%).

Evaluating horses when ridden also gave the opportunity to observe the behaviour of horses during tacking-up and mounting. It has previously been shown that abnormal behaviour during tacking-up and/or mounting is frequently due to anticipation of musculoskeletal pain when ridden [31,32]. In a small proportion of horses with girth aversion behaviour, equine gastric ulcer syndrome may be the underlying cause [33]. In the current study, five horses showed abnormal behaviour during tacking-up ± mounting, four of which had gait dysfunction at the PPE and one that developed lameness after purchase. Observation of abnormal behaviour during tacking-up or mounting is therefore an indicator that something is not right and provides potentially valuable additional information.

Repetitive grinding of the teeth has previously been observed in horses undergoing investigation of poor performance [34]. When underlying musculoskeletal pain was abolished using diagnostic anaesthesia, together with a change in saddle when required, teeth grinding stopped. Three of the four horses in the current study exhibiting teeth grinding had an unsuccessful outcome. A fourth stopped this behaviour after extensive musculoskeletal treatments, including surgery, and a major change in management. Teeth grinding should therefore not be ignored.

The presence of a crooked tail occurs more frequently in lame versus non-lame horses and has been associated with hindlimb lameness, epaxial muscle hypertension, and lumbosacroiliac joint region pain [35]. The degree of crookedness is often more severe in ridden horses compared to horses evaluated trotting in hand in straight lines (as observed in H1), especially when turning in the direction of the crooked tail, as well as in canter compared with trot. The presence of a crooked tail is therefore worth noting and is part of the RHpE.

The results of the current study reinforced how lameness and other gait dysfunctions may be more evident during ridden exercise than trotting in straight lines in hand [5,36] or on the lunge, with all horses considered as high risk having exacerbation of gait abnormalities when ridden. Historically, there has been a focus on the detection of lameness rather than looking at horses’ gait patterns and quality under a variety of circumstances, including canter. Canter dysfunction is usually the result of musculoskeletal pain [34,37]; is often most obvious during ridden exercise, as seen in the current study; and can have a major influence on the overall performance of sports horses. Saddle slip is usually an indicator of hindlimb lameness and is often worse in canter than in trot; therefore, its presence potentially highlights gait dysfunction [2,28].

The use of the RHpE was a valuable addition to gait assessment. Although the most sensitive and specific threshold value for determining the presence of musculoskeletal pain is a score of 8/24 [38], most non-lame sports horses that perform optimally have scores of 0–4/24 [16,17,18,19,26], while higher scores are often associated with inferior performance [21,22,23,24,25]. Therefore, if a horse has a score > 4/24, it should be carefully scrutinised. Although a Canadian-based study indicated that rider skill did not influence the display of lameness in non-lame and lame horses [39], a more recent study showed that a skilled rider may conceal signs of lameness in some horses and improve gait quality compared to a horse’s normal rider [15]. However, on average, RHpE scores were similar for both riders, so a skilled rider could not conceal behavioural signs of discomfort. In the current study, low RHpE scores facilitated making some decisions to recommend purchase, whereas a high RHpE often highlighted the significance of other clinical observations, resulting in advising against a purchase. These data therefore highlight the value of ridden exercise as part of PPEs.

One horse (H9) had only been in light work for six weeks prior to the PPE after a break of approximately six months, allegedly for genuine reasons unrelated to the horse. The horse was considered an intermediate risk for purchase (suitable with reservations) but was bought nonetheless. Ridden behavioural problems rapidly ensued. There are always risks of horses changing in movement patterns and behaviour with a different rider, a change in tack, or alterations in many aspects of management. However, this horse epitomises the hazards of performing PPEs in horses that are not in full work and have had an unexplained break.

One horse (H11) was being treated with altrenogest at the time of the PPE because she had displayed behaviour interpreted as ‘moody’ during ridden exercise. It seems unlikely that her behaviour was related to oestrus cycles. A recent study demonstrated that of 94 mares presented for investigation of abnormal behaviour related to oestrus cycles, none had a final diagnosis of an oestrus-cycle-related problem [40]. Lameness and internal medicine problems, or a combination thereof, predominated. Twenty mares were being treated with altrenogest at the time of investigation, with the persistence of behavioural abnormalities suggesting that suppression of oestrus did not alter symptoms. It is therefore unlikely that administration of altrenogest will conceal abnormal ridden behavioural signs at a PPE.

The results of the current study also highlighted the importance of the evaluation of muscle development patterns; the reaction to systematic palpation of the cervicothoracic, thoracolumbosacral, and pelvic regions; and a horse’s reaction when stimulated to flex, extend, and rotate the thoracolumbosacral and pelvic regions. The strong association between the presence of palpable abnormalities of the thoracolumbosacral and pelvic regions during static examination and lameness, canter dysfunction, and RHpE scores during ridden exercise adds further evidence that abnormal behaviours during ridden exercise are pain-associated.

There are few other studies describing the outcome of PPEs of sports horses [41,42,43]. In a study of 134 horses in California, USA, 37% of horses were considered ‘serviceably sound’, with a lower proportion in competition horses (24–33%, depending on work discipline) compared with pleasure riding horses (48%) [41]. In a second study from California, 43% of 510 horses were considered non-lame [42]. In a more recent United Kingdom study involving a mixture of low-level competition and pleasure riding horses, 43% of 133 horses did not have ‘prejudicial findings’ [43]. In the current study, which included overt lameness and other gait dysfunction in either trot or canter, 32% of horses were considered an acceptable risk for purchase. The results of the four studies are remarkably similar, but no follow-up data for the previous studies were available [41,42,43].

This is only a small study and therefore the results of the statistical analyses should be interpreted with care. However, obvious differences were seen between the ‘Reasonable Risk’ and higher risk groups. Moreover, the small number of horses facilitated accurate acquisition of long-term follow-up information. The study was restricted to dressage, showjumping, and eventing horses in the United Kingdom; whether similar results would be obtained in other geographical locations and with other work disciplines and pleasure riding horses merits further investigation. The study was limited to the observations of a single clinician who was familiar with all the purchasers, most of whom were ambitious amateur riders. A single clinician provides consistency but may have introduced inherent bias. There were no agents involved in the sales. The study was restricted to dressage, showjumping, and event horses that were intended to compete under the auspices of official governing bodies of the sports, and whose performance records could therefore be followed using objective unbiased data. The odds of a ‘Reasonable Risk’ horse fulfilling its performance expectations were 96 times higher than those for a horse in the higher risk categories. This suggests that the clinical findings identified during the ridden exercise/RHpE application were accurate predictors of long-term performance. However, there are multiple reasons why a horse may not compete (for example, change in the rider’s circumstances, unrelated injuries) and it cannot be concluded with certainty that it was always related to the findings of the PPE. Horses may have been downgraded and competed at unofficial competitions. It is also important to recognise that the performance of the risk classification was assessed against each horse’s competition outcome and did not provide deterministic prediction of future musculoskeletal health or injury.

## 5. Conclusions

In conclusion, there appears to be strong evidence that there are benefits to including ridden exercise and the application of RHpE during PPEs of prospective sports horses. Inclusion of ridden exercise allows for the observation of behaviour during tacking-up and mounting, the identification of the presence of saddle slip, and highlights gait and behavioural abnormalities not seen in hand and on the lunge.

## Figures and Tables

**Table 1:** Previous uses of 25 horses undergoing pre-purchase examinations, with the intended use by the purchaser, horse descriptions, and potentially clinically relevant results of static examinations. H = horse number; WBL = Warmblood; ISH = Irish Sports Horse; TB = Thoroughbred. G = gelding; M = mare; GP = general purpose (including unaffiliated competition); BE = British Eventing; FEI = Fédération Equestre Internationale.

H	Previous Use	Ultimate Intended Use	Age Years	Breed	Sex	Static Examination
1	Potential Event Horse	Eventing FEI 1*	5	WBL	G	Poor development of the thoracolumbar epaxial muscles and pelvic and hamstring musculature. Right hindquarter marginally less well-muscled than the left.
2	Potential Event Horse	Eventing FEI 1*	5	WBL	G	Exaggerated response to pressure applied over the tubera sacrale.
3	Eventing BE 90	Eventing BE Novice	7	TB	G	Thoracic sling muscles and dorsocaudal neck muscles poorly developed.
4	Dressage Novice	Dressage Grand Prix	5	WBL	M	Left front digit had more foot and pastern lateral to a line that perpendicularly bisected the metacarpal and fetlock regions compared with medially. Medial bulb of heel slightly displaced proximally. Mild mediolateral imbalance (higher laterally). Both front feet had a low heel. Tubera sacrale higher than withers.
5	Potential Dressage	Dressage Grand Prix	4	WBL	M	Overweight; body condition score 7.5/9.
6	Potential Eventing	Eventing BE 100	6	WBL	G	Left hindquarter slightly less well-muscled than right; left tuber sacrale lower than right. Cervical, thoracolumbosacral region, and epaxial muscles poorly developed; concavities in cranial saddle region; prominence of summits of the lumbar spinous processes.
7	Potential Showjumping	Showjumping 1.20 m	5	WBL	M	Left hindquarter slightly less well-muscled than right; asymmetrical front feet—right front taller and more upright than left front; right front foot mediolateral imbalance; unshod behind and misshaped hoof capsules.
8	Showjumping 90 cm	Showjumping 1.10 m	8	Cross	M	No significant findings.
9	GP	Eventing BE 100	6	ISH	G	Thoracolumbar and pelvic regions poorly muscled, resulting in abnormal prominence of the tubera sacrale (only in work for 6 weeks).
10	GP	Eventing BE 100	5	ISH	G	Ventral neck muscles overdeveloped; right hindquarter less well-muscled.
11	Dressage Elementary (treated with altrenogest because ‘moody’)	Dressage Grand Prix	6	WBL	M	Abnormal hair wear in right caudal saddle region, suggestive of saddle slip to right; concavity in longissimus dorsi muscle immediately cranial to this; considerable increased tension in epaxial muscles in right caudal thoracic and lumbar regions; hyperreactive to pressure applied over tubera sacrale before and after exercise, after > before; similar response left and right sides.
12	Hunting	Eventing FEI 1*	8	WBL	G	Shiverer in both hindlimbs (hyperflexion and abduction); hindquarters and hamstrings not symmetrically developed, right < left.
13	Dressage Novice	Eventing BE Novice	5	WBL	G	Foot of the left hindlimb was long and the pastern foot axis was broken backwards.
14	Raced as 2-year-old	Eventing BE Novice	5	TB	G	Tubera sacrale higher than the withers. Right hindquarter less well-muscled than the left. Neck was set low. Exaggerated response to pressure applied over the tubera sacrale. Tension in the right epaxial muscles from the caudal thoracic to sacral regions. Effusion in the right femoropatellar and medial femorotibial joints. When urinating, the horse did not extend the right hindlimb caudally. Reluctant to perform thoracic lifts and kicked. Front feet asymmetrical. Toes of the hind feet were squared.
15	Eventing FEI 2*	Eventing FEI 5*	7	WBL	G	Metacarpal regions of both forelimbs were offset laterally relative to the central limb axis of the antebrachium and carpus.
16	Dressage Novice	Eventing FEI 2*	6	WBL	G	Reactive to palpation of left brachiocephalicus muscle; resented deep palpation in left cranial saddle region. Would not repeatedly flex, extend, or rotate lumbosacral region; became agitated; turned head to girth, ears back, and fidgeted (before and after exercise).
17	GP	Eventing BE 100	5	ISH	M	No significant abnormalities.
18	GP	Eventing BE Novice	6	ISH	G	Pigeon-toed; metacarpal regions set on lateral relative to central limb axis of antebrachium and carpus; tubera sacrale higher than withers.
19	GP	Eventing BE 100	6	WBL	M	No significant abnormalities.
20	Dressage Advanced Medium	Dressage Grand Prix	9	WBL	G	Right hindquarter slightly less well-muscled than left. Lumbar and pelvic muscles poorly developed; hypertension in epaxial muscles in lumbar region, right > left; hair wear in caudal saddle region, right > left, and further away from midline, suggestive of saddle slip to right.
21	Dressage Elementary (had not competed for 7 months)	Dressage Prix St George	9	WBL	G	Lumbar epaxial muscle hypertension, left and right sides. Resented stimulation to extend lumbosacral region; exaggerated response to pressure over tubera sacrale; thoracolumbosacral epaxial muscles poorly developed (saddle had tight tree points & dry underneath cranial aspect of saddle after ridden exercise).
22	Showjumping 1 m	Showjumping 1.10 m	8	Cross	G	Hypertension in lumbar epaxial muscles, left = right.
23	Eventing (British pony team)	Eventing Novice	12	Pony	G	Slightly lordotic thoracolumbar posture.
24	Dressage Novice	Dressage Prix St George	7	WBL	G	Resented distal limb flexion of right forelimb a lot; poor mediolateral balance in right front foot.
25	Showjumping 1 m	Eventing Novice	7	Cross	G	Very reactive to pressure over tubera sacrale; limited flexibility in thoracolumbosacral region; hypertension in lumbar epaxial muscles; neck poorly muscled.

**Table 2:** Summary of observations during the dynamic examinations of 25 horses undergoing pre-purchase examinations. H = horse number; NAD = no abnormality detected; RHpE = Ridden Horse Pain Ethogram score (maximum 24); NA = not applicable. Lameness grades (0–8): 1 = just discernible; 2 = mild; 3 = mild–moderate; 4 = moderate.

H	In Hand	Lunge Soft Surface	Lunge Firm Surface	Ridden	RHpE Score
1	Mild outward rotation of the heel of each hind foot and slight hock oscillation; tail to left	Longer steps than when ridden	Longer steps than when ridden	Crooked tail, head behind vertical, head up & down, spooked, teeth grinding a lot; short stepping	4
2	NAD	NAD	NAD	Low-grade hock oscillations in walk, left hind > right hind; initially repeatedly swished its tail, head behind vertical, with an unsteady head carriage and head tilt; ill-fitting saddle	4
3	NAD	Lame right front 3/8 on left rein	NAD	Mouth open in canter	2
4	NAD	Croup high in canter; tail held slightly to left; slightly greater trunk lean on the right rein compared with the left rein	NAD	Could engage hindlimbs better in canter; worked in a rather fixed and rigid way—needed to ‘work over the back’ better—may be helped by better fitting saddle; in canter right, forelimb step length needed to increase (possibly saddle related)	1
5	NAD	NAD	NAD	NAD	2
6	Lame left hind 1/8; left hind flexion → grade 3/8 lame left hind	Less well balanced on left rein compared with right rein; more inwards trunk lean; left hindlimb crossed under trunk when protracted on left rein; bilateral hindlimb toe drag	Better than on soft	Head behind vertical all the time in trot; could not get horse to lengthen its neck frame and move more fluently through thoracolumbosacral region with greater range of motion; needed more hindlimb engagement in canter	3
7	Lame left hind 1/8	NAD	NAD	No lameness seen or felt when ridden, active and willing; canter similar on both reins and of good quality; opened mouth & increased rein tension (‘gobby’) in downward transitions	2
8	NAD	NAD	NAD	NAD	3
9	NAD	Bucked going into canter on both the left and right reins; ears back	NAD	Back of saddle bounced dorsoventrally and rider was positioned on caudal third of saddle; stumbled twice behind; lacked hindlimb impulsion going uphill in trot but moved better going downhill (ridden in a field with a gradient); bucked and kicked out going into left canter, and tail swished repeatedly in canter	4
10	Lame right hind 2/8; uncomfortable during right front flexion and transiently lame right front afterwards 2/8; slightly positive left hind flexion 1/8	Right hind crossed under trunk during protraction on right rein	Right hind crossed under trunk during protraction on right rein	Unsteady head position; right hind crossed under trunk during protraction on right rein	3
11	Subtle left hind lameness 1/8; no swing of tail reflecting reduced range of motion of thoracolumbosacral region; repeatedly slammed down left hindlimb during flexion—because of reluctance to stand on right hind or uncomfortable with left hind flexion or training problem; increased left hind lameness to 3/8	Bilateral hindlimb toe drag, right = left; reduced range of motion of thoracolumbosacral region and lack of swing of tail; canter on forehand with stiff hindlimb gait; hindlimbs placed rather close together; left rein shortened cranial phase of step right hind and slightly irregular rhythm; 2/8 lame right hind; right rein better balanced and more fluid	Hindlimb toe drag	Head behind vertical all the time to variable extent; head tilt repeatedly, especially in 10 m diameter circles to left in rising trot; saddle slip to right, left rein > right rein and canter > trot; hindlimb gait less restricted in rising trot than sitting trot; not overtly lame	4
12	After each forelimb flexion test, jumped into trot with irregular hindlimb rhythm; flexion left hind—2/8 lame left hind; flexion right hind—2/8 lame right hind	Left rein—lame right front 3/8	Left rein lameright front 3/8	Tight tree points of saddle; lame right front 3–4/8 on left and right reins going around periphery of field; in 10 m circles, 4/8 lame right front on left rein; no change in treeless saddle	6
13	Low grade forelimb and hindlimb ataxia	Bilateral hindlimb toe drag, inside hindlimb > outside hindlimb; poor hindlimb engagement in downward transitions from canter to trot	NAD	Bilateral hindlimb toe drag, right = left; inside hindlimb crossed under trunk during protraction; unsteady head position	5
14	Bilateral hindlimb toe drag, left > right	Reduced range of motion of thoracolumbosacral region; bilateral hindlimb toe drag and lack of hindlimb impulsion; canter short stepping, stiff and stilted	NAD	Reduced range of motion of thoracolumbosacral region; bilateral hindlimb toe drag and lack of hindlimb impulsion; lame left hind 2/8 on left rein and right hind 3/8 on right rein; canter was short stepped, stiff, and stilted; saddle slip to right, canter > trot; tacking-up and ridden—teeth grinding	16
15	Short stepping forelimbs; flexion left hind—lame 3/8 left hind	Short stepping forelimb gait in trot & canter; right canter crooked; head tossing	Shortened forelimb step length; lame left front 1/8, consistent on left rein and episodic on right rein; head tossing	Hunched back when mounted; unsteady head carriage; canter croup high and crooked to the right; saddle slip to right on left rein; when worked in a more correct outline, lame left forelimb 3/8 on left rein; canter restricted, lacked hindlimb impulsion, and was not forward enough; tension ++	4
16	Stiffer turning to left in small circles	Exaggerated contractions of epaxial muscles, canter > trot	NAD	Extended thoracolumbosacral region when walking first few steps after mounting; marked change in demeanour, balance, and overall movement; ears back, front of head behind vertical, unsteady head, intense stare, head tilt, teeth grinding, tail swishing, mouth open to variable extent, spontaneously broke from trot to canter, and repeatedly above the bit in transitions to right canter; in sitting trot, reduced range of motion of thoracolumbosacral region and increased mouth opening; downward transitions from trot to walk or canter to trot on forehand, with more mouth opening; head more unsteady in leg yield and shoulder-in	7
17	NAD	NAD	Intermittent lame steps right front 3/8 on the right rein; on the left rein, occasional lame steps right front 1/8	Much more difficult to turn to right than to left; never going forwards; unwilling; stumbled badly in front once	4
18	Lame left hind 2/8	NAD	NAD	Lame left hind 4/8 on left and right reins; lame right front 3/8 on left rein; canter lacked suspension; disunited repeatedly on right rein; more comfortable on right rein in trot compared with left rein	6
19	‘Winged’ both forelimbs; ‘footy’	NAD	Shorter forelimb steps than on soft	Bilateral hindlimb toe drag, right = left; marked saddle slip to right; much shorter stepping on grass than in arena; bucked in trot canter transitions	5
20	Short stepping gait; hindlimbs ‘stiff’	Lack of hindlimb engagement and impulsion	Lack of hindlimb engagement and impulsion	‘One paced’ on the forehand, lacked hindlimb engagement and impulsion in trot and canter; saddle slip to the right, canter > trot	9
21	Lame left hind 1–2/8; left hind flexion → lame left hind 4/8	Toe drag of inside hindlimb, which crossed under the trunk during protraction; reduced range of motion of the thoracolumbosacral region in canter; exaggerated contractions of epaxial muscles	Left rein lame—left hind 2/8	Mounting—yanked downwards with head and neck; left rein lameleft hind 3/8; constantly chomping on bit; did not want to properly flex lumbosacral joint and engage hindlimbs and lacked hindlimb impulsion, canter > trot; canter poorly balanced—croup high; canter pirouettes—struggled to maintain rhythm—hindlimbs placed closely together	8
22	Stiff hindlimb gait	Lacked hindlimb impulsion and engagement	Stiff	Lacked hindlimb impulsion and engagement; saddle slip to left on right rein; struggled with repeated trot-walk–trot transitions, repeatedly ‘jumping’ into trot—reflecting lack of hindlimb power—and tended to step short behind in downward transitions; rather stiff-legged hindlimb gait in canter with lack of flexion at lumbosacral joint; canter induced rotation of rider’s pelvis and generated large impulse through rider’s back	7
23	Short stepping; right hindlimb toe drag	Lacked hindlimb impulsion and engagement; canter on forehand ++	Very unbalanced & short stepping	Jumped into trot; bent excessively to right with excessive rein tension on left side; poor hindlimb impulsion and engagement; right canter on forehand > left, and generated large impulse through rider’s back; ‘braced’ thoracolumbosacral region in canter-trot transitions and head raised; lame left hind 3/8 in medium trot and 10 m diameter circles	6
24	Sustained right forelimb lameness 5/8 after distal limb flexion; proximal limb flexion left hind—lame left hind 3/8; proximal limb flexion right hind—lame left hind 2/8	Right forelimb lameness 2/8 on the left and the right reins	Right forelimb lameness—2/8 on the left and the right reins; shortened forelimb step length	Not performed	NA
25	Lame left front 1/8; flexion right front—lame right front 4/8; flexion left hind—lame 1/8 left hind; flexion right hind—lame right hind 2/8	Bilateral hindlimb toe drag, right > left; short stepping forelimb gait; looked to outside right rein > left	Lame left front—2/8 on left rein; lame right front—4/8 on right rein	Not performed	NA

**Table 3:** Outcome of pre-purchase examinations of 25 horses examined consecutively during a period of six months, with long-term performance 3.5 to 4 years after examination. H = horse number; BE = British Eventing.

H	Recommendation	Long-Term Outcome
1	Reasonable risk	Bilateral hindlimb proximal suspensory desmopathy and lumbosacroiliac joint region pain. After successful treatment the horse changed ownership, is used for general purpose riding, and has stopped grinding teeth.
2	Reasonable risk	Comminuted articular fracture of the proximal phalanx of the right forelimb.
3	Reasonable risk	Eventing BE 90.
4	Reasonable risk	Advanced medium dressage, mean score 68%.
5	Reasonable risk	Advanced medium dressage, mean score 67%.
6	Reasonable risk	Eventing BE 100.
7	Reasonable risk	Showjumping 1.10 m.
8	Reasonable risk	Junior showjumping.
9	Suitable with reservations but purchased	Challenging behaviour when ridden; hindlimb proximal suspensory desmopathy and lumbosacroiliac joint region pain; bilateral front foot pain & forelimb proximal suspensory desmitis.
10	Suitable with reservations—insurance company placed exclusions; not purchased	General purpose riding.
11	Suitable with reservations; not purchased	Did not compete for 18 months; then competed advanced medium, mean score 70%.
12	High risk; not purchased	Did not compete in affiliated competitions.
13	High risk; not purchased	Did not compete in affiliated competitions.
14	High risk; not purchased	Retired.
15	High risk; not purchased	Did not compete in affiliated competitions.
16	High risk; not purchased	Sold to another client; returned 6 weeks later because of dangerous behaviour when ridden; did not compete in affiliated competitions.
17	High risk; not purchased	Did not compete in affiliated competitions.
18	High risk; not purchased	Pleasure riding.
19	High risk; not purchased	Pleasure riding.
20	High risk; not purchased	Continued in competition; progressed to Prix St Georges, mean score 65%.
21	High risk; not purchased	Did not compete for one year; then competed for two years at medium and advanced medium, mean score 66%.
22	High risk; not purchased	Did not compete in affiliated competitions.
23	High risk; not purchased	Did not compete for one year; then was downgraded to BE 100 (under 18), competing four times and three times only in successive years; then downgraded to BE 90, but failed to complete four events.
24	Advised against purchase (examination not completed)	Did not compete for six months; then resumed competition for two years in Novice dressage, mean score 68%.
25	Advised against purchase (examination not completed)	Lost to follow-up; did not compete in affiliated competitions.

## Data Availability

Anonymised raw data are available upon reasonable request.
